# Patients with Mild COVID-19 Symptoms and Coincident Pulmonary Embolism: A Case Series

**DOI:** 10.5811/cpcem.2020.7.48254

**Published:** 2020-07-20

**Authors:** Joshua W. Joseph, Jonathan C. Roberts, Cheri N. Weaver, Jonathan S. Anderson, Matthew L. Wong

**Affiliations:** * Beth Israel Deaconess Medical Center, Department of Emergency Medicine, Boston, Massachusetts; † Beth Israel Lahey - Milton Hospital, Department of Emergency Medicine, Milton, Massachusetts

**Keywords:** Coronavirus, pulmonary embolism, emergency medicine, coagulation

## Abstract

**Introduction:**

Frequent thrombotic complications have been reported in patients with severe coronavirus disease 2019 (COVID-19) infection. The risk in patients with mild disease is unknown.

**Case Report:**

We report a case series of three individuals recently diagnosed with COVID-19, who presented to the emergency department with chest pain and were found to have pulmonary emboli. The patients had mild symptoms, no vital sign abnormalities, and were negative according to the pulmonary embolism rule-out criteria.

**Conclusion:**

This suggests that patients with active or suspected COVID-19 should be considered at elevated risk for pulmonary embolism when presenting with chest pain, even without common risk factors for pulmonary embolism.

## INTRODUCTION

Severe infection with coronavirus disease 2019 (COVID-19) has been associated with coagulopathy, with complications ranging from a high rate of pulmonary embolism (PE) in intubated patients, to an increased frequency of premature stroke in young patients. However, the prevalence of clinically significant thrombotic complications in patients with milder symptoms is less clear, and the relative risk imparted by COVID-19 compared to other thrombotic risk factors is unknown. We report a case series of three young patients with confirmed COVID-19 and PE, who presented to the same small, suburban emergency department (ED) over a one-week period. The ED sees an average of 32,000 visits per year, and had approximately 200 confirmed cases of COVID-19 by the time of the last case. The patients had previously been diagnosed with COVID-19, and due to their relatively mild symptoms (including no exertional symptoms and no desaturation), they had been discharged home after ED visits in the previous two weeks. None of the patients had pre-existing comorbidities for venous thromboembolism. We explore their presentations in detail, in order to alert clinicians to the heightened risk of PE in all patients with COVID-19, not only those with critical presentations.

## CASE SERIES

### Case 1

Patient 1 was a 40-year-old man who presented to the ED with mild left-sided chest pain. He had been seen nine days earlier due to fever and cough, and was diagnosed with COVID-19. However, during that visit, he had no shortness of breath, no significant decrease in oxygenation while ambulating, a normal sinus rhythm electrocardiogram (ECG) with no significant ST-segment changes, and was discharged home on precautions. Since his prior visit, he reported no immobility or significant change in daily activities, and he had no significant past medical history and took no medications. Repeat EKG demonstrated no ST-segment changes. The emergency physician caring for the patient was concerned that COVID-19 could be a risk factor for PE, and ordered a D-dimer, which was elevated at 4489 nanograms per milliliter (ng/mL) (reference range 0–499 ng/mL). Troponin and brain natriuretic peptide (BNP) levels were normal. A computed tomography angiogram (CTA) demonstrated bilateral pulmonary emboli and bilateral lower lobe ground-glass opacities consistent with COVID-19 pneumonia. He was discharged on a course of rivaroxaban.

### Case 2

Patient 2 was a 48-year-old man who presented with right-sided chest pain, which was sharp in character and pleuritic. He reported no accompanying dyspnea or worsening of the pain with exertion. His ECG demonstrated normal sinus rhythm without significant ST-segment changes. He reported a past medical history of gout, but was on neither prophylactic treatment nor active treatment for a flare in the months prior to presentation. He had been seen 14 days prior, during which he had presented with similar symptoms, but also with an accompanying fever and dyspnea. During that visit, he underwent a CTA which showed multifocal ground-glass infiltrates, consistent with COVID-19 infection, and was discharged home on isolation precautions.

During his return visit, he underwent D-dimer testing, which was elevated at 2183 ng/mL (reference range 0–499 ng/mL). A CTA demonstrated a right upper segmental PE, multiple subsegmental pulmonary emboli, and progression in the size of ground-glass infiltrates (which were not associated with vascular filling defects). He was briefly admitted to the medical service, and discharged after a two-day admission on a course of apixaban.

### Case 3

Patient 3 was a 47-year-old woman who presented with left-sided chest pain, which was pressure-like in character and non-pleuritic. She noted some worsening of her symptoms with exertion, but the pain was also present at rest. Her ECG showed sinus rhythm without significant ST-segment changes, and an initial troponin was negative (<0.01 ng/mL). She had been seen nine days previously, with cough and dyspnea, and had undergone an evaluation including ECG and chest radiograph, and discharged home on precautions with a presumptive diagnosis of COVID-19. This was confirmed on outpatient testing two days later.

Relative to her initial presentation, she reported that the chest pain she was experiencing was new, but the sensation of dyspnea and the frequency of her cough had lessened significantly. She reported no significant change in activity while at home, and reported no hormone use or other risk factors for PE. A D-dimer was drawn and elevated at 5821 ng/mL (ref: 0–499 ng/mL). CTA was performed and demonstrated emboli throughout the right upper segmental branch and bilateral lower lobe segmental and subsegmental branches. Due to her clot burden, she was admitted to the medical service and started on apixiban. An inpatient echocardiogram demonstrated no evidence to suggest cor pulmonale, and she was discharged after two days.

**CPC-EM Capsule**

What do we already know about this clinical entity?

Coronavirus Disease 2019 (COVID-19) has been associated with coagulopathy in severe cases, leading to high rates of pulmonary embolism among hospitalized patients.

What makes this presentation of disease reportable?

We report several patients with COVID-19 who developed significant pulmonary emboli, despite having mild symptoms of both COVID-19 infection and pulmonary embolism.

What is the major learning point?

These cases suggest that COVID-19 should be treated as an independent risk factor for pulmonary embolism.

How might this improve emergency medicine practice?

Clinicians should have a high index of suspicion for pulmonary embolism in patients with COVID-19 infection who present with chest pain.

## DISCUSSION

Critically ill patients with COVID-19 and acute respiratory distress syndrome have been observed to have a high frequency of PE, as well as diffuse intravascular coagulation.^1^–^3^ Several mechanisms have been proposed for these findings, including inflammatory cytokine production, vascular endothelial disruption within the lungs, and hyaline microemboli formation, which may be complementary factors. This multifactorial coagulopathy likely has an additive effect to existing risk factors for PE in the critically ill, which include immobility, invasive procedures, respiratory failure, and mechanical ventilation.^4^–^5^ Accordingly, some authors have proposed using markers of coagulation, such as D-dimer, platelet count, and partial thromboplastin time as markers of disease severity.^6^–^8^

Extrapulmonary vascular complications of COVID-19 have also been reported, including large-vessel strokes in young patients, and portal venous and mesenteric arterial thrombosis.^9^–^10^ However, these thrombotic complications have not clearly correlated with the severity of patients’ respiratory or systemic COVID-19-related symptoms. Troublingly, in the case series of large-vessel stroke reported by Oxley et al, two of the five patients reported no antecedent respiratory or systemic symptoms to suggest COVID-19 infection.^9^

The patients in our case series are notable because despite having pulmonary emboli, their symptoms of both COVID-19 and PE were relatively mild, and none reported periods of immobility or other clear antecedent risk factors. This is particularly concerning in light of the fact that these patients were initially judged to have a low risk of PE via the pulmonary embolism rule-out criteria (PERC) [Table ]. The PERC rule, introduced by Kline et al, is a well-validated decision tool for screening patients at low risk for PE, with the goal of reducing unnecessary CT imaging by avoiding the D-dimer test and its high rate of false positives.^11^ The rate of PE among patients who are very low risk per the PERC rule is estimated to be less than 2%; thus, many clinicians use the rule in lieu of D-dimer screening for low-risk patients. Its use is widespread throughout emergency medicine.^12^

While our findings represent a relatively small case series, when viewed in the larger context of coagulopathy seen in patients with COVID-19, they suggest that clinicians may need to view a diagnosis or presumed diagnosis of COVID-19 as an independent risk factor for PE, for which the PERC rule cannot be used in lieu of D-dimer testing. More research is needed to examine the ultimate validity of the PERC rule in this population. Considerable debate exists over the use of prophylactic anticoagulant use in patients with COVID-19, with recommendations depending on the degree of associated coagulopathy and inpatient status.^13^–^15^ However, we do not believe that there are data to support such a recommendation for outpatients without either clear evidence of thrombosis or until we have a better understanding of the true prevalence of thrombosis in COVID-19.

## CONCLUSION

Our case series demonstrates a concerning frequency of pulmonary embolism in otherwise healthy patients presenting with mild symptoms of COVID-19 and chest pain. More research is needed to determine whether specific subpopulations with COVID-19 are at increased risk of PE. However, clinicians may need to treat COVID-19 as an independent risk factor for PE in patients presenting with chest pain, and tailor their diagnostic heuristics accordingly, using a D-dimer and Wells’ score rather than the PERC rule.

## Figures and Tables

**Table:** Clinical characteristics of three patients presenting with COVID-19 and coincident pulmonary embolism.

Patient	1	3	4
Age	40	48	47
Sex	Male	Male	Female
Chief complaint	Chest pain	Chest pain	Chest pain
Medical history	None	Gout	Hypertension, Migraine, Anxiety
Risk factors for pulmonary embolism	None	None	None
Medications	None	Colchicine (episodic, not at time of diagnosis)	Amitriptyline, amlodipine, hydrochlorothiazide, lisinopril
Initial visit signs and symptoms of COVID-19	Cough, fever	Dyspnea, fever	Dyspnea
ECG (rhythm)	Sinus	Sinus	Sinus
ECG (ST-segment changes)	T-wave flattening (nonspecific)	None	None
Signs and symptoms of pulmonary embolism	Chest pain	Chest pain	Chest pain
Negative by PERC Criteria	Yes	Yes	Yes
WBC (4.0 – 11.0 k/μL)	6.0 k/μL	11.1 k/μL	10.7 k/μL
Troponin (<0.01 ng/mL)	<0.01 ng/mL	<0.01 ng/mL	<0.01 ng/mL
D-dimer (0–499 ng/mL)	4489 ng/mL	2183 ng/mL	5821 ng/mL
BNP (0–125 pg/mL)	<5.0 pg/mL	Not measured	48.8 pg/mL
Location of clot	Right upper, right middle and bilateral lower lobe lobar pulmonary arteries	Proximal right upper lobe segmental pulmonary artery, subsegmental right upper lobe pulmonary arteries	Right upper lobe and bilateral lower lobe segmental and subsegmental branches
Disposition and outcome	Discharged from ED on rivaroxaban	Admitted for two days, discharged on apixiban	Admitted for two days, discharged on apixiban

*ECG*, electrocardiogram; *PERC*, Pulmonary Embolism Rule-out Criteria; *WBC*, white blood cell count; *k*, thousand; *μL*, microliter; *ng*, nanogram; *mL*, milliliter; *BNP*, B-type natriuretic peptide; *pg*, picogram; *ED*, emergency department.
